# Individuality in Evolution

**Published:** 1911-03

**Authors:** John C. Warbrick

**Affiliations:** Chicago


For Texas Medical Journal.

### Individuality in Evolution.

BY JOHN C. WARBRICK, M. D., CHICAGO.

May we not consider the human species as resulting from the physical forces of its environment. Some conceptions of the working laws of development are so widespread among those who have not made some special study of the subject as well as those who have to point out that we living beings are not altogether clay in the hands of the potter's environment. Individuality has governed the course of the streams of development for ages, although they have all flowed in their course of selection as streams of water flow according to the natural constraint of their banks.

By the individuality of a person or of an animal is meant all that determines its specific characters and mode of action, according to present and past environment as recognized at all times.

Some people inherit a strong individuality that at once distinguishes them from all others, while one child may develop an individuality entirely different to that of another, although born of the same parents.

The qualities included are those which distinguish one individual from another in choice, tastes, desires, etc., for people, as is only natural, differ in many ways as in temperament, occupations, diet and habits as a matter of choice or from constitutional tendencies, while such differences are noticeable in animal life to a more marked degree. There is also the natural selection of animals in regard to climate, some preferring a colder climate to that of a warmer one, as those with hair and those without it, the former naturally adapting themselves to the colder regions, the latter to the warmer zones.

Many examples of each of these might readily be given, as for instance the polar bears and the reindeer inhabit the colder zones, while the kangaroo is found always in the warmer parts of the globe, along with the giraffe, thus adapting themselves to climatic conditions on the earth for sustenance according to the natural laws of environment.

Each country and climate, then, have animals that are habitants of them according to the physical conditions of the earth, and such are always found there, and in no other region. If transported to another zone, they would not find the conditions natural nor conducive to their welfare, and thus in time death would result.

Individuals have the power by their environment to determine what course they intend to pursue, and thus the various tastes have produced a rarety in the forms of life and living of men. Our ancestors did not choose to remain in England but came to the United States and Canada, while the Pilgrim Fathers chose to leave their homes to found a new Commonwealth amidst hardships by courage and perseverance.

A man can not only choose his own surroundings, but he can modify and improve and establish changes therein and fit them to himself in a large degree.

Thus it is the man often of the greatest intelligence who advances ahead of his fellows. Hence his marked superiority to the animal in this and other ways. He explores new continents and opens them up for commerce. He goes to the Arctic and Antarctic regions, and by taking the furs of animals he makes his environment quite adaptable to himself.

Thus the world has been created out of elements of various kinds so that man and beast in different countries and climates can adapt themselves to the varied conditions of living and temperature.

